# Hippocampus-Avoidance Whole-Brain Radiation Therapy Is Efficient in the Long-Term Preservation of Hippocampal Volume

**DOI:** 10.3389/fonc.2021.714709

**Published:** 2021-08-19

**Authors:** Ilinca Popp, Alexander Rau, Elias Kellner, Marco Reisert, Jamina Tara Fennell, Thomas Rothe, Carsten Nieder, Horst Urbach, Karl Egger, Anca Ligia Grosu, Christoph P. Kaller

**Affiliations:** ^1^Department of Radiation Oncology, Medical Center, Faculty of Medicine, University of Freiburg, Freiburg, Germany; ^2^Department of Neuroradiology, Medical Center, Faculty of Medicine, University of Freiburg, Freiburg, Germany; ^3^Medical Physics, Department of Radiology, Medical Center, Faculty of Medicine, University of Freiburg, Freiburg, Germany; ^4^Department of Oncology and Palliative Medicine, Nordland Hospital, Bodø, Norway; ^5^Department of Clinical Medicine, Faculty of Health Sciences, University of Tromsø, Tromsø, Norway; ^6^German Cancer Consortium (DKTK), Partner Site Freiburg, German Cancer Research Center (DKFZ), Heidelberg, Germany

**Keywords:** hippocampus, atrophy, WBRT (whole-brain radiation therapy), cognitive function, MRI

## Abstract

**Background and Purpose:**

With improved life expectancy, preventing neurocognitive decline after cerebral radiotherapy is gaining more importance. Hippocampal damage has been considered the main culprit for cognitive deficits following conventional whole-brain radiation therapy (WBRT). Here, we aimed to determine to which extent hippocampus-avoidance WBRT (HA-WBRT) can prevent hippocampal atrophy compared to conventional WBRT.

**Methods and Materials:**

Thirty-five HA-WBRT and 48 WBRT patients were retrospectively selected, comprising a total of 544 contrast-enhanced T1-weighted magnetic resonance imaging studies, longitudinally acquired within 24 months before and 48 months after radiotherapy. HA-WBRT patients were treated analogously to the ongoing HIPPORAD-trial (DRKS00004598) protocol with 30 Gy in 12 fractions and dose to 98% of the hippocampus ≤ 9 Gy and to 2% ≤ 17 Gy. WBRT was mainly performed with 35 Gy in 14 fractions or 30 Gy in 10 fractions. Anatomical images were segmented and the hippocampal volume was quantified using the Computational Anatomy Toolbox (CAT), including neuroradiological expert review of the segmentations.

**Results:**

After statistically controlling for confounding variables such as age, gender, and total intracranial volume, hippocampal atrophy was found after both WBRT and HA-WBRT (*p* < 10^−6^). However, hippocampal decline across time following HA-WBRT was approximately three times lower than following conventional WBRT (*p* < 10^−6^), with an average atrophy of 3.1% *versus* 8.5% in the first 2 years after radiation therapy, respectively.

**Conclusion:**

HA-WBRT is a therapeutic option for patients with multiple brain metastases, which can effectively and durably minimize hippocampal atrophy compared to conventional WBRT.

## Introduction

Cerebral radiation therapy (RT) is a central pillar in the treatment of brain metastases (1). For patients with multiple metastases, whole-brain RT (WBRT) is a common treatment option, as it was shown to significantly improve distant intracerebral tumor control and reduce the neurological death rate compared to local therapies alone (2). However, with increased survival due to improved systemic and supportive therapies, reported neurocognitive deficits following cerebral irradiation and in particular WBRT have gained substantial importance (3, 4). More specifically, WBRT is associated with an increased risk of cognitive dysfunction and decline in quality of life (3–6), with numerous prior studies having deemed RT-induced hippocampal damage the most important culprit (7–11). Cognitive decline can be observed as early as 6 weeks after WBRT (3, 5) and appears to predominantly involve verbal memory (3, 12, 13).

Hippocampus-avoidance WBRT (HA-WBRT) selectively restricts the radiation dose in the hippocampal region with the intention of preserving cognitive functions. It is generally considered a safe method, with a low risk of hippocampal and peri-hippocampal relapse (10, 14–16). The protective effect of HA-WBRT on the hippocampi has been and is currently still being investigated in prospective clinical trials, but mainly indirectly by means of neurocognitive testing. In the single arm RTOG 0933 trial (9) and in the randomized phase III NRG Oncology CC001 trial (10), a reduction in neurocognitive decline was observed following HA-WBRT compared to conventional WBRT. The evaluation of neurocognitive functions in these trials could only be reliably performed for a maximum of 6 months following RT, although more than 50% of patients were still alive after this point (10). High death rates and noncompliance with neurocognitive testing may thus hinder a comprehensive long-term evaluation using neurocognitive testing as a proxy of hippocampal damage. Furthermore, distinguishing tumor fatigue and declining physical health from a specific RT-related hippocampal neurocognitive failure remains challenging.

A more direct measurement of hippocampal cellular loss after irradiation can be the assessment of changes in hippocampal volume as a function of dose and time. Hippocampal neuronal and volume loss have been systematically linked to cognitive decline, independently of concomitant neuropathological diseases (17–19). However, at present, it is unknown to which extent and over which period of time HA-WBRT can prevent hippocampal cellular loss compared to conventional WBRT. To close this gap, we retrospectively identified WBRT and HA-WBRT patients longitudinally monitored with magnetic resonance (MR) imaging and extracted hippocampal volume as a morphological parameter to elucidate both immediate and long-term effects of WBRT and HA-WBRT and to determine the extent to which HA-WBRT can prevent hippocampal atrophy compared to conventional WBRT over time.

## Materials and Methods

### Patient Sample

**Figure 1:**
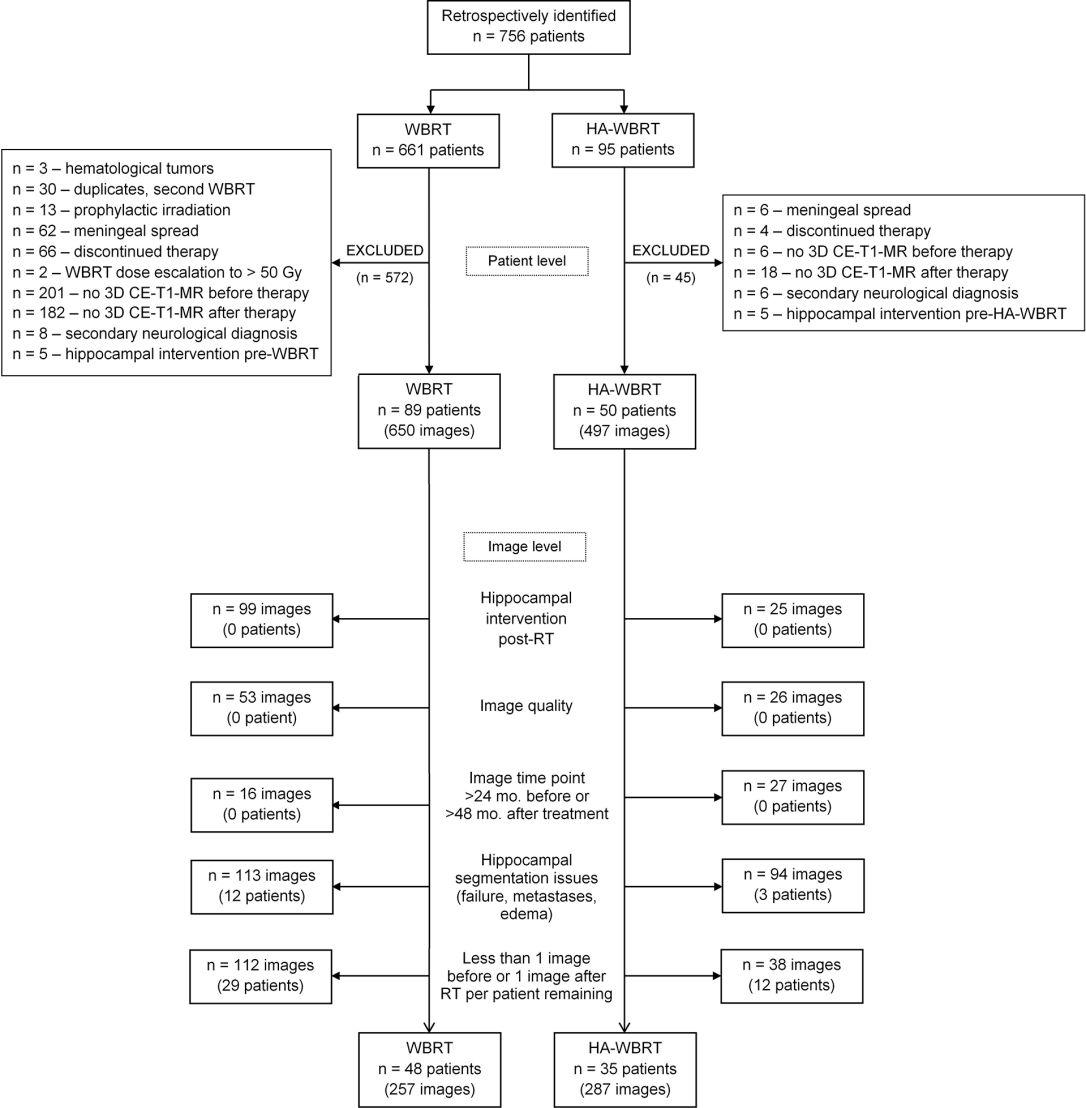
Flowchart of patient selection. CE-T1-MR, contrast-enhanced T1-weighted magnetic resonance; HA-WBRT, hippocampus-avoidance whole-brain radiation therapy; RT, radiation therapy; WBRT, whole-brain radiation therapy.

The current study was approved by the local ethics committee. We used a retrospective longitudinal study design and identified 756 patients having received WBRT or HA-WBRT between December 2003 and December 2016 in the Department of Radiation Oncology of the Medical Center—University of Freiburg. Patients were evaluated with respect to inclusion/exclusion criteria on the patient level and image level, as is specified in the flowchart in **Figure 1**.

Patients were included if they had cerebral metastases of solid tumors, no meningeal spread at the time of WBRT/HA-WBRT, no known central nervous system pathologies accompanied by cognitive deficits or radiological changes (e.g., dementia, stroke, and meningitis), and at least one gross artifact-free three-dimensional (3D) contrast-enhanced sagittal T1-weighted MR (CE-T1-MR) imaging study before and after irradiation. Patients with hippocampal metastases or hippocampal interventions prior to study treatment were not considered suitable for analysis. Hippocampal interventions were defined as hippocampal resections or RT to the head with a total mean hippocampal dose (D_mean_, summed across all RT series) ≥ 3 Gy (equivalent dose delivered in 2 Gy fractions [EQD2, *α*/*β* = 2]) and a total maximal hippocampal dose (D_max_, summed across all RT series) ≥ 14.4 Gy (EQD2, *α*/*β* = 2). The thresholds were set taking into consideration the strictest hippocampal constraints imposed in clinical trials (10, 20, 21).

After this first selection on the patient level, patients were evaluated on the image level. In patients with any further hippocampal interventions (see definition above) after RT, all imaging studies acquired after these interventions were excluded to avoid any bias on the target analyses. Studies lacking the appropriate quality for processing were also excluded. To this end, image quality was assessed by means of the Image Quality Rating (IQR) derived from the brain tissue segmentation using the Computational Anatomy Toolbox (CAT). The IQR metric is a continuous index that scales between 0% and 100% and is graded from A+ to F, which corresponds to an image quality from 100% to 50% (and below), respectively. Images with grades A, B, and C are considered to be of excellent, good, to satisfactory quality, whereas grades D, E, and F denote a sufficient, critical, and unacceptable image quality, respectively. For the present analyses, the threshold for inclusion of individual image studies was set to ≥70%, which corresponds to an image quality of at least C- (satisfactory).

MR imaging at follow-up examinations had been performed every 3 months or as required according to clinical routine. The interval for inclusion of image time points into the present analysis was set to 24 months before and 48 months after RT, in order to account for a maximal general life expectancy of cerebrally metastasized patients (22).

Insufficient quality of the automatic segmentation of the hippocampi with the CAT (see below) and the presence of edema or metastases in the hippocampi as further exclusion criteria were visually checked by an experienced neuroradiologist (AR). Finally, after exclusion of individual imaging studies, only those patients having at least two imaging studies (minimum one study before and minimum one after RT) remained in the analysis.

### Radiation Treatment Planning

Patients underwent RT-planning computer tomography (CT) in thermoplastic mask immobilization (BrainLab, Feldkirchen, Germany). CE-T1-MR and CT images were rigidly co-registered based on mutual information (iPlan RT Image 4.1.1, BrainLab, Feldkirchen, Germany) and served for target volume and organ at risk delineation.

For HA-WBRT, a hippocampus-avoidance region (HAR) was defined as a 7-mm 3D margin around the hippocampus, as described previously (23, 24). The planning tumor volume (PTV) for brain was defined as the whole brain (clinical target volume, CTV) plus 3 mm, excluding PTVs of metastases and the HAR. The prescribed dose for the brain PTV was 30 Gy in 12 fractions, with or without simultaneous integrated boost of 51 Gy or 42 Gy in 12 fractions to the metastases. The hippocampal avoidance was performed according to the constraints of the currently ongoing prospective randomized trial HIPPORAD (NOA-14, ARO 2015-3, DRKS00004598): dose to 98% of the hippocampal volume (D_98%_) ≤ 9 Gy, dose to 2% of the hippocampal volume (D_2%_) ≤ 17 Gy, and D_mean_ ≤ 10 Gy (17). Patients were treated by volumetric modulated arc therapy (VMAT) based on 2–4 arcs.

The WBRT was performed in the majority of cases by conventional two-dimensional planning (98.1%). A minority received CT-based three-dimensional planning (1.9%). The prescribed dose was 35 Gy in 14 fractions in 43.9%, 30 Gy in 10 fractions in 30.8%, 40 Gy in 20 fractions in 11.2%, and other fractionations in 14% of cases.

### Dosimetry and Interfering Events

The D_max_ and D_mean_ applied to the hippocampi and to the whole brain during WBRT and HA-WBRT were extracted from the dose-volume histograms and converted into equivalent doses delivered in 2 Gy fractions (EQD2), considering an *α*/*β* = 2, in order to account for the different prescription doses and fractionations. Previous and subsequent RT to the brain, head or neck structures and their corresponding doses to the hippocampi, as well as hippocampal resections and edema were also documented.

### Image Processing

**Figure 2:**
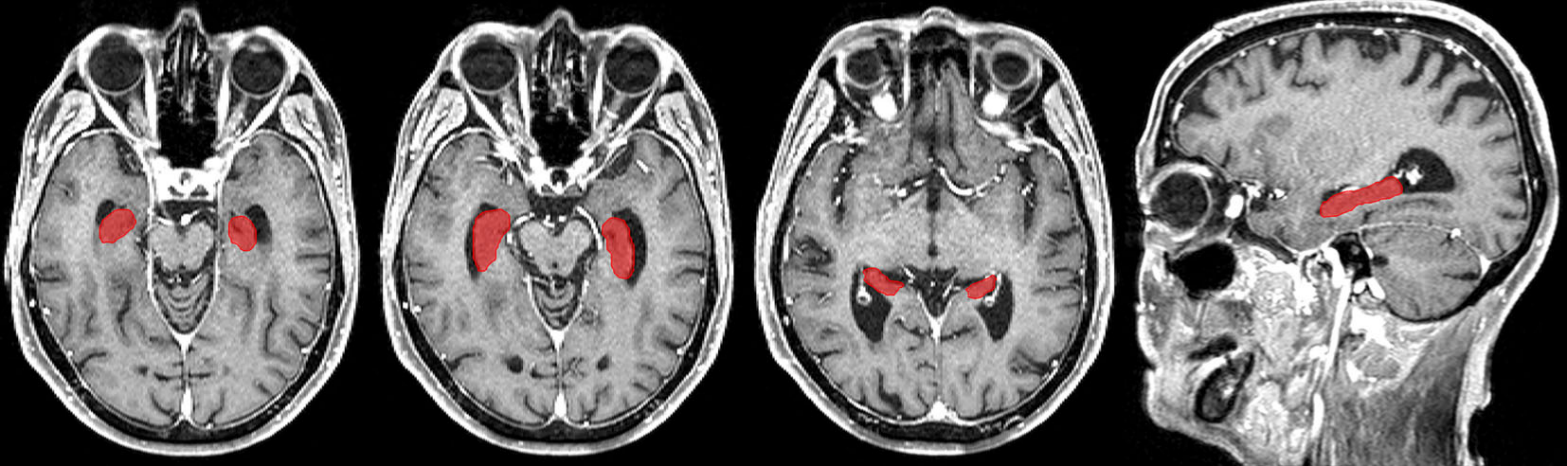
Example of a hippocampus segmentation on CE-T1-MR imaging using the CAT and the *in vivo* high-resolution CoBrA atlas of the hippocampus (26).

CE-T1-MR images were segmented using the CAT (version 12.5, release 1364; http://dbm.neuro.uni-jena.de/cat/) with default parameter settings running in the Statistical Parametric Mapping (SPM, version 12.5; https://www.fil.ion.ucl.ac.uk/spm/software/) software package in Matlab (version 7.14; The Mathworks Inc., Natick, MA). Deformation field parameters for nonlinear normalization into the stereotactic Montreal Neurological Institute (MNI) standard space were computed using the DARTEL (Diffeomorphic Anatomical Registration Through Exponentiated Lie algebra) approach (25) implemented in CAT. Atlas-based segmentation of the hippocampus and resulting hippocampal volumes were computed based on the *in vivo* high-resolution Computational Brain Anatomy (CoBrA) atlas of the hippocampus (26) implemented in CAT. An example of a hippocampus segmentation using CAT is shown in **Figure 2**.

CAT segmentation was found to be reliable and robust compared to the ground truth (27). However, for quality assurance, the automatized segmentation was verified by an experienced neuroradiologist (AR). CE-T1-MR images were evaluated in a 3D reformation with regard to the accuracy of the segmentation of the hippocampi, the total intracranial volume (TIV), and the brain volume. In addition, occurrence of hippocampal or parahippocampal metastases with hippocampal edema was assessed. Imaging studies featuring insufficient hippocampal segmentation accuracy or the presence of metastases and/or edema were excluded from the analysis (see above).

### Statistical Analysis

We hypothesized that treatment with WBRT compared to HA-WBRT leads to a stronger decrease in hippocampal volume across time following RT. Given presumably non-linear patterns of volume change across time, we took advantage of the statistical software R (version 3.4.4 (28)) and the package *mgcv* [version 1.8-31 (29, 30)] for generalized additive mixed modelling (GAMM). Additive modeling fits a smoothing curve on subsegments of the data using regression splines (29–31) and can cope with non-linear patterns without the need of prior knowledge on the exact shape of the function underlying the relationship of interest. Effective degrees of freedom (edf) for the individual model terms are estimated from the data but were in the present analyses restricted by default to *k* = 10 (30). Univariate smooths with thin plate regression splines were used as smoothing functions. Reported model estimates were based on a restricted maximum likelihood (REML) approach.

Hippocampal volume as derived from the CAT segmentation (see above) constituted the dependent variable. Given that the raw hippocampal volume data were substantially correlated between hemispheres (*r* = 0.823) and that no hypotheses were specified for differential effects of RT on left versus right hippocampus, we decided to use the average of the raw hippocampal volumes across hemispheres as dependent variable to reduce the dimensionality of the data.

Therapy group (WBRT vs. HA-WBRT) and time (as continuous measure in months centered at the time point of RT) comprised the independent variables of interest to be modeled as fixed effects. The effects of time and the interaction of time by group were thereby modeled using non-linear smoothing functions.

Age at RT, gender, and TIV constituted nuisance variables that were expected to have a systematic impact on interindividual variation in hippocampal volume but were of no interest and modeled as fixed effects. As an exploratory analysis indicated an expectably strong confound between TIV and gender [*r* = 0.602; see also (32)], we decided to orthogonalize the two variables by regressing TIV on gender and to further use the residuals as gender-adjusted index of interindividual variation in TIV.

The longitudinal design with different number of observations for individual patients irregularly spaced in time and measured on different MR scanners was taken into account by modeling variations between patients and MR scanners as random intercepts.

Taken together, our target analysis on the differential time course of changes in hippocampal volume following WBRT vs. HA-WBRT thus comprised a GAMM model with the dependent variable volume, the three fixed effects of interest (time, group, and the interaction between group and time), three fixed effects for nuisance variables (age at RT, gender, and gender-adjusted TIV), and two random effects (patient and MR scanner). By restricting the maximum possible number of smoothing functions to *k−*1 = 9 for the effects of age and the interaction between age and group (see above), the possible total number of individual fixed-effects parameters including the intercept ranged between 7 and 23 (for potentially resulting *k−*1 numbers of smoothing functions between 1 and 9, respectively). Furthermore, given a total of 544 valid data points (see below), this corresponded to a ratio of at minimum ≥ 23 to at maximum ≤ 77 observations per fixed-effect parameter, which was hence sufficient for a valid model estimation and not prone to overfitting (30).

## Results

### Final Patient Sample

On the patient level, 617 out of the initially identified 756 suitable patients were considered unsuitable and excluded from further analysis (see **Figure 1** for details). Starting in 2012, all patients in our department with multiple metastases of solid tumors, without (peri)hippocampal metastases and eligible for CT-based RT-planning, underwent HA-WBRT with or without simultaneous integrated boost. The remaining patients (with hematological malignancies, prophylactic or repeated WBRT, meningeal spread, or extremely poor prognosis without possibility of follow-up) were treated with conventional WBRT, but were removed from further analysis as per set exclusion criteria. In contrast, all patients before 2012 consistently received WBRT. Thus, the two resulting cohorts were chronologically shifted, but with a low risk of biased selection.

After this first selection on the patient level, 139 patients remained, cumulating in 1,147 CE-T1-MR imaging studies that were further evaluated on the image level. This resulted in the exclusion of another 603 studies and 56 patients (see **Figure 1** for details).

The final data set comprised 544 CE-T1-MR imaging studies (WBRT, *n* = 257; HA-WBRT, *n* = 287) of 83 patients (WBRT, *n* = 48; HA-WBRT, *n* = 35) acquired on 16 different MR scanners (**Figure 1**). The utilized MR scanners were sufficiently overlapping between groups to allow for including MR scanners into the model as a random effect. The individual number of included imaging studies before and after RT ranged between 1 and 10 and between 1 and 20 per patient, respectively.

An overview on the selected patients’ demographic and clinical characteristics is provided in **Table 1**. Patients in the two groups showed imbalances regarding age (*p* = 0.049) and gender (*p* = 0.061), which were statistically accounted for in the target analyses on hippocampal volume (see below). Groups did not significantly differ in the patients’ TIV (*p* = 0.894) and individual maximum follow-up time covered post RT (*p* = 0.974). Primary tumors comprised 12 different etiologies (breast cancer, gastrointestinal tumors, germinal tumors, gynecologic tumors, malignant melanoma, small and non-small cell lung cancer [NSCLC], pancreas tumors, renal cell carcinoma, salivary gland carcinoma, sarcoma, and carcinoma of unknown primary). Considering recent improvements in systemic therapies for NSCLC and melanoma through the introduction of immune checkpoint inhibitors and third-generation tyrosine kinase inhibitors, we decided to evaluate the frequency of these primary tumors versus the remaining etiologies. The analysis indicated a percentage of approximately 50% NSCLC/melanoma, similar in both RT groups (*p* = 0.406). A detailed listing of all applied systemic therapy agents can be found in **Supplementary Table S1**. Finally, there was a significantly higher proportion of HA-WBRT patients (51.1%) than WBRT patients (20.8%) with a history of additional radiotherapy (radiosurgery and stereotactic fractionated radiotherapy) with very low-dose hippocampal exposure (*p* = 0.004; **Table 1**).

**Table 1:** Clinical details of selected patients.

Patient Characteristics	WBRT	HA-WBRT	Differences between groups (test, *p*-value)
Age (years), median, range	59, 34–80	54, 33–84	Mann–Whitney *U* = 1,053.5, *p* = 0.049
Gender (no.), male/female	25/23	11/24	*χ*^2^ = 3.52, *p* = 0.061
Total intracranial volume before RT (ml), median, range	1,438, 1,240–1,715	1,435, 1,209–1,934	Mann–Whitney *U* = 855, *p* = 0.894
Hippocampal volume before RT (ml), median, range	3.37, 2.36–4.03	3.24, 2.45–4.25	Mann–Whitney *U* = 908, *p* = 0.536
Follow-up time (months), median, range	6.9, 2.5–39.1	7.8, 1.8–47.0	Mann–Whitney *U* = 836, *p* = 0.974
Additional low-dose RT hippocampal exposure (before and/or after WBRT/HA-WBRT) with total D_mean_ < 3 Gy and D_max_ < 14.4 Gy (no.): yes/no	10/38	18/17	*χ*^2^ = 8.48, *p* = 0.004
Primary tumor (no.): melanoma+NSCLC/other	23/25	20/15	*χ*^2^ = 0.69, *p* = 0.406

RT was performed according to the prescribed doses, achieving a median D_mean_ (EQD2 *α*/*β* = 2) for the whole brain of 39.4 Gy (range 37.5–40.0 Gy) in the WBRT group and 34.9 Gy (range 33.4–39.7 Gy) in the HA-WBRT group. WBRT in the selected patients was performed exclusively by conventional two-dimensional planning. Thus, D_mean_ and D_max_ (EQD2 *α*/*β* = 2) for both hippocampi were identical to the whole-brain doses and ranged between 37.5 and 40.0 Gy, with a median of 39.4 Gy. In the HA-WBRT group, D_mean_ for the left hippocampus ranged between 5.8 and 8.4 Gy, with a median of 6.8 Gy, while D_max_ ranged between 12.5 and 24.1 Gy, with a median of 15.7 Gy (EQD2 *α*/*β* = 2). For the right hippocampus, D_mean_ was in median 6.7 Gy (range 5.5–9.2 Gy), while D_max_ was 14.8 Gy (range 11.3–21.8 Gy).

Finally, controlling for selection bias, we compared characteristics of patients included in the present analyses to those of the excluded patients (cf. flowchart in **Figure 1**), revealing a significant difference for age (median of 54 vs. 63 years, respectively; *p =* 4.34 × 10**^−^** ^5^) but neither for gender (male vs. female, *n* = 36/47 vs. *n* = 316/327; *p = .322*) nor for type of primary tumor (NSCLC/melanoma vs. other, *n* = 43/40 vs. *n* = 293/350, *p = .*283). The significantly higher median age in the group of excluded patients was concomitant with a significantly shorter median survival time (3.8 vs. 12.7 months; *p =* 4.17 × 10**^−^** ^13^).

### Target Analysis on RT-Induced Hippocampal Atrophy

A generalized additive mixed model (GAMM) of the differential time course of changes in hippocampal volume between groups revealed a significant main effect of time (*F* = 10.19, *edf* = 2.63, *p* = 7.48 × 10**^−^** ^7^) and a significant interaction of time by group (*F *= 8.44, *edf* = 4.04, *p* = 1.14 × 10**^−^** ^7^), whereas the simple effect of group was not significant (*t* = −0.05, *p* = 0.957). Fixed effects of nuisance variables age (*t* = −3.77, *p* = 1.19 × 10**^−^** ^4^), gender (*t* = 4.61, *p* = 5.19 × 10**^−^** ^6^), and gender-adjusted TIV (*t* = 11.78, *p* < 10**^−^** ^16^), as well as random effects of patient (*F* = 19.57, *edf* = 74.13, *p* < 10**^−^** ^16^) and MR scanner (*F* = 11.96, *edf* = 7.46, *p* = 1.25 × 10**^−^** ^4^), also reached significance. Model validation indicated no relevant deviations from the underlying assumptions (**Supplementary Figure S1**).

**Figure 3:**
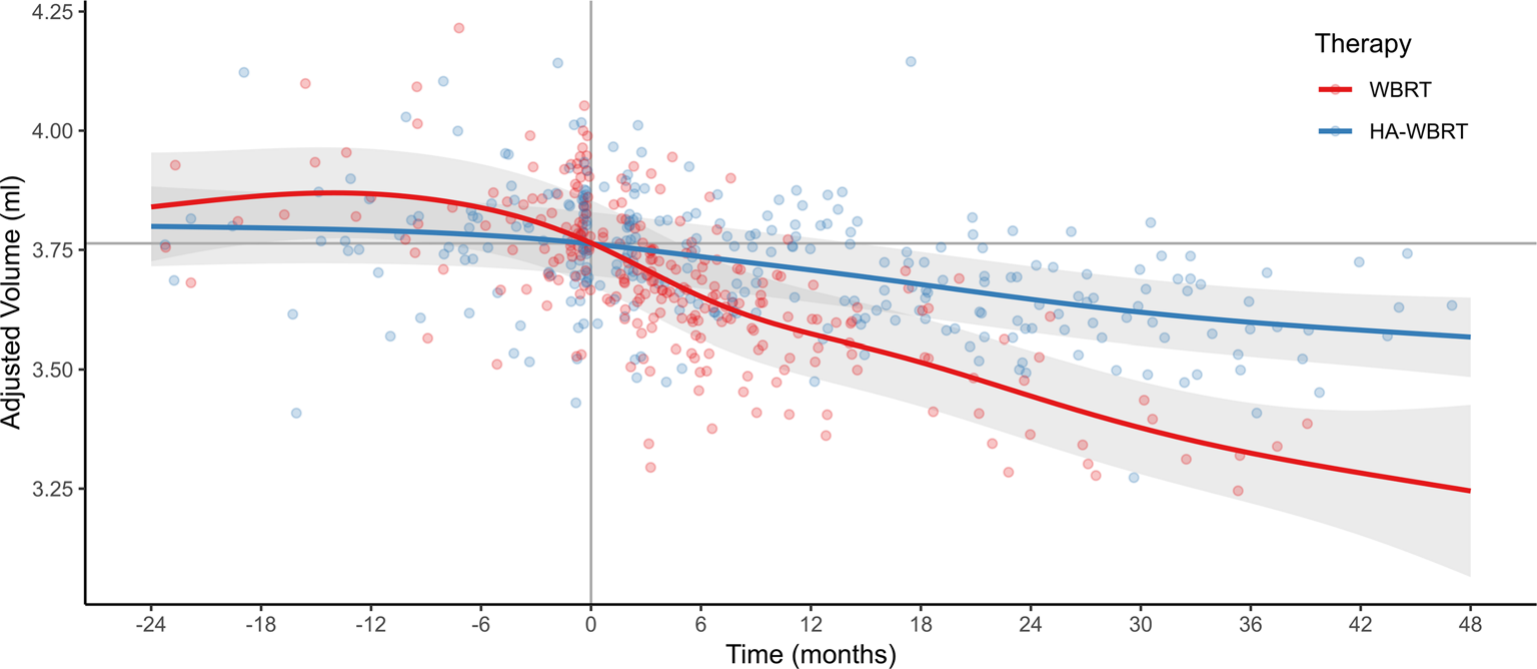
Evolution of hippocampal atrophy 24 months before and 48 months after WBRT versus HA-WBRT (averaged for left and right hippocampus) across time (with the gray vertical line denoting the time of RT and the gray horizontal line depicting the average hippocampal volume at the time of RT as reference). The two treatment groups, WBRT and HA-WBRT, show significantly distinct time courses. Dots represent individual data points, and bands represent standard errors. Adjusted volume refers to the estimated marginal means after accounting for variation of nuisance variables (effects of age, gender, TIV, patient, and MR scanner). The number of patients with adequate imaging studies still in follow-up was *n* = 49 at 6 months, *n* = 29 at 12 months, *n* = 14 at 24 months, and *n* = 5 at 36 months. HA-WBRT, hippocampus-avoidance whole-brain radiation therapy; WBRT, whole-brain radiation therapy.

As can be seen in **Figure 3**, treatment with WBRT was associated with a significantly steeper atrophy of hippocampal volume compared to treatment with HA-WBRT. In the WBRT patients, the estimated average hippocampal volume loss after 6, 12, 24, and 48 months (with time of RT as reference) comprised −0.113 ml (95% prediction interval [−0.288, +0.063]), −0.190 ml [−0.369, −0.012], −0.320 ml [−0.505, −0.136], and −0.519 ml [−0.873, −0.165]. This was equivalent to a volume loss at 6, 12, 24, and 48 months of −3.0% [−7.8%, +1.82%], −5.1% [−10.0%, −0.1%], −8.5% [−13.9%, −3.1%], and −13.8% [−24.7%, −2.9%]. In the HA-WBRT patients, the estimated average hippocampal volume loss after 6, 12, 24, and 48 months (with time of RT as reference) was −0.027 ml [−0.158, +0.104], −0.055 ml [−0.187, +0.077], −0.116 ml [−0.252; +0.019], and −0.196 ml [−0.358, −0.033]. This was equivalent to a volume loss at 6, 12, 24, and 48 months of −0.7% [−4.2%, +2.8%], −1.5% [−5.0%, +2.1%], −3.1% [−6.8%, +0.6%], and −5.2% [−9.75%, −0.7%].

The predicted hippocampal volume decline following WBRT was therefore approximately three times higher in the first 2 years posttreatment than following HA-WBRT.

In contrast, volume changes 24, 12, and 6 months before the time of RT as reference comprised +0.076 ml ([−0.148, +0.299]; +2.0% [−3.8%, +7.8%]), +0.103 ml ([−0.082, +0.288]; +2.7% [−2.0%, +7.5%]), and +0.074 ml ([−0.105, +0.253]; +2.0% [−2.7%, +6.6%) in the WBRT patients and +0.037 ml ([−0.128, +0.201]; +1.0% [−3.3%, +5.3%]), +0.028 ml ([−0.109, +0.166]; +0.8% [−2.9%, +4.4%]), and +0.018 ml ([−0.144, +0.151]; +0.5% [−3.0%, +4.0%]) in the HA-WBRT patients, respectively. That is, predicted hippocampal volume changes before RT were substantially lower, not significantly different from zero, and comparable between groups.

### Supplementary Analyses

We computed several control analyses, which are in brief reported below. (i) To answer the question whether hippocampal atrophy over time was significant on the level of the individual treatments, we computed two GAMMs on the effect of time separately for the two groups (each including only the effect of time, but neither group nor the interaction between time and group; plus fixed nuisance effects of age, gender, gender-adjusted TIV, and random intercepts for patient and scanner). Results confirmed significant changes in hippocampal volume across time for both WBRT (*F* = 28.52, *edf* = 4.47, *p* < 10**^−^** ^16^) and HA-WBRT (*F* = 13.51, *edf* = 2.18, *p* = 1.91 × 10**^−^** ^7^). (ii) Furthermore, to more directly consider the time of RT as the actual onset of the observed hippocampal atrophy, we extended the original GAMM of our target analysis by the factor pre/post and accordingly centered the continuous variable time pre RT to −24 months and restricted the post RT data to the first 24 months. The model hence comprised the fixed effects of time (continuous), group, time point (pre vs. post RT), and their two-way and three-way interactions, as well as the fixed nuisance effects of age, gender, gender-adjusted TIV, and random intercepts for patient and scanner. Results revealed the critical three-way interaction of time by group by pre/post (*F* = 9.94, *edf* = 2.00, *p* = 6.13 × 10**^−^** ^5^), thus statistically confirming that the differential effects in hippocampal volume decline between groups and across time were indeed manifest post RT (see also **Figure 3**). (iii) Finally, given the chronological shift between the data acquisitions in the two treatment groups and potentially confounding changes in secondary systemic therapies for melanoma and NSCLC tumors, we conducted a control analysis explicitly testing whether the interaction effect of time by RT group on the evolution of hippocampal atrophy was differentially driven by primary tumor (melanoma and NSCLC *vs.* other). That is, we extended our target analysis by the factor primary tumor type, thus resulting in model with fixed effects for time, group, primary tumor, and their two-way and three-way interactions, as well as the fixed nuisance effects of age, gender, gender-adjusted TIV, and random intercepts for patient and scanner. However, neither the critical three-way interaction of time by RT group by primary tumor nor any lower-order effects of primary tumor were significant (all *p* > 0.266).

## Discussion

The current study used retrospective longitudinal analysis of hippocampal volume as a direct morphological marker to determine the impact of moderate RT doses on the hippocampus in the context of whole-brain irradiation. In a sample of patients with multiple (>3) brain metastases closely followed-up with serial MR imaging, we found significant hippocampal atrophy over time after both WBRT and HA-WBRT, with considerably lower atrophy rates following the latter. To our knowledge, this is the first study to show the differential time course of the effects of WBRT with and without hippocampal avoidance on hippocampal volume.

### Hippocampal Atrophy Following Radiotherapy

For the fractionated, partial brain RT of primary brain tumors, Seibert et al. (33) similarly measured the hippocampal volume in 52 patients before and 1 year after treatment. The authors found a significant reduction in volume after high-dose RT (D_mean_ > 40 Gy), but not after low-dose RT (D_mean_ < 10 Gy). Our results substantially extend these findings by longitudinally demonstrating the impact of moderate-dose RT (median D_mean_ < 40 Gy) and low-dose RT (median D_mean_ < 7 Gy) on the hippocampus as applied in the WBRT and HA-WBRT group, respectively. Moreover, the here observed annual atrophy rate of approximately 5% in the first 2 years after WBRT clearly exceeds the reported mean annualized rates of 3.5%–4% for patients with Alzheimer’s disease (17). Furthermore, these values also surpass those observed in elderly patients experiencing worsening cognitive decline (17).

In normal aging, hippocampal atrophy relative to the rest of gray matter is reported to begin after the ages of 63 in men and 67 in women (34) and increase to an estimated annual decline rate of 1.7% after the age of 80 (17). In the HA-WBRT cohort, the median age was 54, with only 14% of patients over the age of 63. Thus, the significant hippocampal atrophy of 1.6% per year in the first 2 years after RT seems higher than what would be expected for this age group (34) and contrasts notably the lack of volume change in the 2 years prior to HA-WBRT in the same patients. Since hippocampal D_mean_ for HA-WBRT was generally below 7 Gy EQD2 *α*/*β* = 2 and a significant atrophy was noticed only after this intervention, our data suggest a possibly high hippocampal radiosensitivity to lower doses, similar to the observations of Mizumatsu et al. in animal studies (7). These results are in line with the data of Nagtegaal et al., who found a dose-dependent increase in hippocampal age of 2–20 years and a hippocampal volume loss rate of 0.16%/Gy in 33 patients having undergone RT for grade II–IV glioma (35). Compared to this, the atrophy rate obtained for our HA-WBRT cohort was slightly higher (1.6% versus 0.16 × 6.8 Gy = 1.1%). Whether and to which extent additional low-dose hippocampal exposure (with a total D_mean_ < 3 Gy, EQD2 *α*/*β* = 2) from previous and subsequent radiotherapies may have contributed to the hippocampal atrophy in the HA-WBRT group is unclear and has to be explored in future trials.

Although hippocampal volume assessment may not be sensitive to all forms of neurodegeneration, hippocampal volume loss has been systematically associated with cognitive decline in dementia, with significantly higher atrophy rates in patients showing clinical worsening (17, 18). Following RT, hippocampal atrophy in general (36, 37) and the inhibition of neurogenesis in the neural stem cell niche found in the subgranular zone of the dentate gyrus in particular are considered to be responsible for memory impairment (7, 38). Although data on the persistence of human hippocampal neurogenesis in adults is controversial (39, 40), sparing of the hippocampi in RT planning appears to be clinically relevant and effective in preventing cognitive deterioration (9, 10, 41).

In this respect, various hippocampal constraints have been considered safe for irradiation. In the NRG Oncology CC001 trial (10) and the preceding single-arm RTOG 0933 trial (9), 100% of the hippocampus did not exceed a dose of 9 Gy (6.5 Gy EQD2 *α*/*β* = 2), and the hippocampal D_max_ did not exceed 16 Gy in 10 fractions (14.4 Gy EQD2 *α*/*β* = 2). Dosimetric analyses performed after stereotactic fractionated RT for benign or low-grade adult brain tumors revealed an equivalent dose of 7.3 Gy in 40% of the bilateral hippocampi (D40%, EQD2 *α*/*β* = 2) as cutoff for the occurrence of long-term cognitive impairment (11). Another clinical trial exploring hippocampal sparing prophylactic cranial irradiation in patients with small-cell lung cancer limited hippocampal D_mean_ to 8 Gy in 10 fractions (5.6 Gy EQD2 *α*/*β* = 2) (21). In the ongoing prospective randomized HIPPORAD trial (NOA-14, ARO 2015-3, DRKS00004598), the hippocampal constraints include D_98%_ ≤ 9 Gy (6.2 Gy EQD2 *α*/*β* = 2), D_2%_ ≤ 17 Gy (14.5 Gy EQD2 *α*/*β* = 2), and an aimed D_mean_ ≤ 10 Gy (7.1 Gy EQD2 *α*/*β* = 2) (20). The equivalent dose applied in our HA-WBRT-cohort was therefore in alignment with these data and could be considered sufficient for neurocognitive protection. Consistent with clinical observations, our results showed that HA-WBRT prevents considerable hippocampal volume loss compared to conventional WBRT. Evaluating the time course of hippocampal decline in both groups, the atrophy rate was highest within the first six months following RT and decreased thereafter. Despite this deceleration across time, the differential three-times lower atrophy rate for HA-WBRT persisted over a time frame of 4 years after RT and remained significant after accounting for patient age, gender, and TIV.

### Limitations

Because of the retrospective study design, a major limitation of the study is that clinically relevant neurocognitive functional parameters have not been systematically assessed so that a direct link between clinical neurocognitive outcome and hippocampal atrophy after RT could not be established. Furthermore, the final data set was also considerably smaller compared to the initially identified patient list. While the stringent patient selection may impact the generalizability of results, this was necessary to ensure homogeneity and a correct data interpretation. The analysis of excluded patients showed no major discrepancies in gender and tumor type, but revealed lower survival rates corresponding to older age and poorer prognosis. The selected cohort could thus be not representative for all cerebrally metastasized patients, but for the population most eligible for HA-WBRT.

In spite of this selection, there are still several medical conditions that may also influence the size of the hippocampus with increasing age (e.g., cardiovascular disease, obesity, diabetes, hypertension, anxiety, or clinical depression) (42). However, considering the chronological shift of the two groups, the risk of biased selection based on potentially relevant comorbidities was minimized.

Another possible confounder is represented by the cancer and treatment-related neurocognitive dysfunction, reported in the majority of cancer patients and colloquially known as “chemobrain” (43, 44). Although differences in applied systemic therapies did not seem to influence the degree of hippocampal atrophy in our cohort, differences in type and duration of systemic therapies due to the chronological shift may still have had disparate effects on hippocampal volume, as preclinical data suggest a negative impact on neurological pathways and cognition. In particular, hippocampal neurogenesis seems to be inhibited by a wide range of chemotherapeutic agents, including the commonly used cisplatin, oxaliplatin, and paclitaxel (45–47). Morphological alterations and synaptic dysfunction were also noticed in the treatment with certain immune, targeted, and hormone therapies (48–51). However, clinical data on these effects are extremely scarce. While some MRI studies suggest reductions of hippocampal volume in patients receiving systemic treatment (52), others do not (53). Moreover, to our knowledge, the influence of dose and duration of the applied therapies was not investigated as of now. These particularities may thus constitute unknown confounders, which were not systematically documented and could not be included in the present analysis. Given its detailed documentation and prospective design, these interfering aspects will be further explored in the ongoing randomized HIPPORAD trial (20).

Finally, the allowed small RT doses to the hippocampus (in total D_mean_ < 3 Gy and D_max_ < 14.4 Gy, EQD2 *α*/*β* = 2) during additional interventions (radiosurgery and stereotactic fractionated radiotherapy) before and after study treatment (WBRT vs. HA-WBRT) may have also had an effect on hippocampal volume. However, this affected the HA-WBRT group substantially more than the WBRT group, so that the results of our target analysis showing a preservation of hippocampal volume after HA-WBRT remain valid. Similarly, the prescribed RT regimens were not uniform, but heterogeneous in both the WBRT and the HA-WBRT group, with higher doses applied to the whole brain in the WBRT group. While the effect of the whole brain dose on hippocampal volume independently of hippocampal dose is not known, this difference may have also had an impact on the dynamics of hippocampal atrophy. However, the variation in hippocampal dose between groups (difference in median hippocampal D_mean_ of 32.6 Gy) was higher by one order of magnitude compared to the hippocampal dose variation within the individual groups (D_mean_ range of 2.5 Gy and 3.7 Gy for WBRT and HA-WBRT, respectively) and to the whole-brain dose variation between groups (difference in median D_mean_ of 4.5 Gy). Therefore, we do not expect a significant impact on the results of our target analysis.

## Conclusion

The current study shows that HA-WBRT may effectively and durably minimize hippocampal damage compared to conventional WBRT, achieving a threefold reduction in atrophy over a time frame of 4 years following irradiation. To which extent low or cumulative radiation doses over time or applied systemic therapies may also have a significantly negative impact on hippocampal volume and hippocampal-related cognition is still unclear and warrants further investigation in clinical trials.

## Data Availability Statement

The metadata supporting the conclusions of this article will be made available by the authors, without undue reservation. Requests to access the datasets should be directed to Ilinca Popp (ilinca.popp@uniklinik-freiburg.de).

## Ethics Statement

The studies involving human participants were reviewed and approved by Ethics Committee of the Albert-Ludwigs-University Freiburg. Written informed consent for participation was not required for this study in accordance with the national legislation and the institutional requirements.

## Author Contributions

Study design: AG, CK, IP, KE, and CN. Patient treatment according to protocol: IP, AG, TR, KE, and HU. Data collection: IP. Quality assurance: AR and KE. Analysis and interpretation of data: CK and IP. Provision of data analysis tools: EK and MR. Writing of the manuscript: IP and CK. Revision of the manuscript and input: AR, JF, TR, CN, HU, KE, and AG. All authors contributed to the article and approved the submitted version.

## Funding

IP was partially funded by the Else Kröner-Fresenius-Stiftung, Germany, within the EXCEL (Excellent Clinician Scientists in Freiburg—Education for Leadership) Program “Immunological Causes and Therapies of Cancer” and by the IMM-PACT-Programme for Clinician Scientists, Department of Medicine II, Medical Center—University of Freiburg and Faculty of Medicine, University of Freiburg, funded by the Deutsche Forschungsgemeinschaft (DFG, German Research Foundation)—413517907. CPK was supported by the BrainLinks–BrainTools Cluster of Excellence funded by the German Research Foundation (EXC 1086). The funding sources had no involvement in the writing of the manuscript or in the decision to submit the article for publication.

## Conflict of Interest

The authors declare that the research was conducted in the absence of any commercial or financial relationships that could be construed as a potential conflict of interest.

## Publisher’s Note

All claims expressed in this article are solely those of the authors and do not necessarily represent those of their affiliated organizations, or those of the publisher, the editors and the reviewers. Any product that may be evaluated in this article, or claim that may be made by its manufacturer, is not guaranteed or endorsed by the publisher.
