# Supplementary material for: Hippocampus-Avoidance Whole-Brain Radiation Therapy Is Efficient in the Long-Term Preservation of Hippocampal Volume
Source: Front Oncol. 2021 Aug 19;11:714709. doi: 10.3389/fonc.2021.714709 (PMC8417356; doi:10.3389/fonc.2021.714709)
Supplement: Supplementary file 1 [file DataSheet_1.pdf]

Supplementary Material

1 Supplementary Figures and Tables

1.1 Supplementary Figure

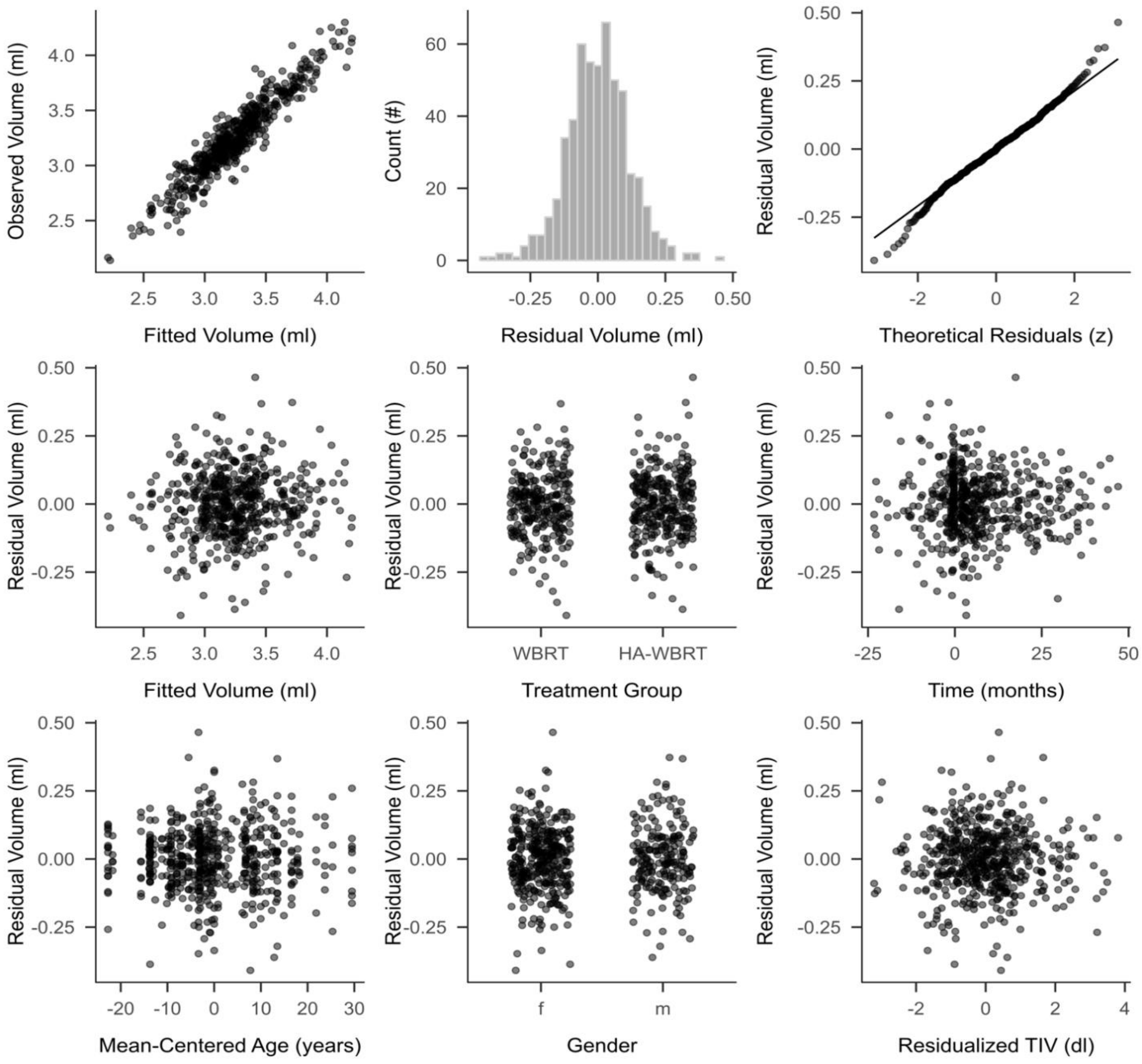

Supplementary Figure 1. Model validation indicated no serious deviations from the underlying assumptions (normality, homogeneity, independence): (a) Observed hippocampal volumes plotted against fitted volumes showed a good model fit with a large amount of explained variance (adjustedR2 = 0.871) and without any systematic structures in the residuals. (b) Normality was assessed by inspection of residuals showing that these followed a normal distribution with (c) only slight deviations from the theoretical distribution in few data points at the margins. (d) Homogeneity was assessed by plotting residuals against fitted hippocampal volumes showing no obvious patterns

and a nearly identical spread of residuals across fitted volumes (except for very few cases with fitted volumes below 2.5 ml). Independence was assessed by plotting residuals against explanatory variables, i.e. (e) RT group, (f) time, (g) age, (h) gender, and (i) TIV. All plots except for time showed nearly identical spreads of residual variance across the spectra of observed values in the individual explanatory variables. For time, a larger spread of residuals was observed for the pre-RT interval, presumably due to the more sparse and less consistent and continuous sampling of MR images before RT. Additionally checking the robustness of the model fit against the observed minor deviations from assumptions of normality, homogeneity, and independence, we computed several control analyses (e.g., excluding data points with theoretical residuals $|z| > 2$ or with fitted volumes < 2.5 ml, restricting the analysis to the time post RT only) which all consistently corroborated the reported results. That is, the present findings can be considered valid and were not driven or distorted by any deviations.

1.2 Supplementary Table

Supplementary Table 1. List of systemic therapies applied before and/or after RT.

**Chemotherapy**	**Immunotherapy**	**Targeted therapy**	**Endocrine therapy**	**Other**
cisplatin	trastuzumab	sunitinib	anastrozole	mitoxantrone
oxaliplatin	bevacizumab	everolimus	letrozole	thalidomide
capecitabine	interferon alpha	erlotinib	exemestane	samarium-153
fluorouracil	cetuximab	gefitinib	tamoxifen	
cyclophosphamide	pertuzumab	afatinib	fulvestrant	
(Nab) paclitaxel	ipilimumab	vemurafenib	goserelin	
vincristine	pembrolizumab	dabrafenib	leuprorelin	
doxorubicin	atezolizumab	palbociclib		
methotrexate		trametinib		
temozolomide		cobimetinib		
cytarabine		lapatinib		
carboplatin				
docetaxel				
vinorelbine				
gemcitabine				
pemetrexed				
dacarbazine				
topotecan				
etoposid				
trofosfamide				
eribulin				
melphalan				
treosulfan				
ifosfamide				
fotemustine				
lomustine				
epirubicin				
irinotecan				
